# Navigation of total knee arthroplasty: rotation of components and clinical results in a prospectively randomized study

**DOI:** 10.1186/1471-2474-12-16

**Published:** 2011-01-15

**Authors:** Jan Schmitt, Carsten Hauk, Heino Kienapfel, Michael Pfeiffer, Turgay Efe, Susanne Fuchs-Winkelmann, Thomas J Heyse

**Affiliations:** 1 Department of Orthopedics and Rheumatology, University Hospital Marburg, Germany; 2 Auguste Viktoria Klinik, Berlin, Germany; 3 Helios Rosmann Klinik, Breisach, Germany

## Abstract

**Background:**

Navigation was introduced into total knee arthroplasty (TKA) to improve accuracy of component position, function and survival of implants. This study was designed to assess the outcome of navigated TKA in comparison with conventional implantation with the focus on rotational component position and clinical mid-term results.

**Methods:**

In a prospectively randomized single-blinded approach, 90 patients with primary gonarthrosis were assigned to three different groups. Thirty patients each were assigned to NexGen LPS without and with navigation (groups 1 and 2), and 30 patients to navigation with the Stryker Scorpio PS (group 3). The navigation system used was the imageless Stryker KneeTrac, version 1.0. Clinical outcome was assessed by a blinded observer applying the Knee Society Score (KSS) and a visual analogue scale (VAS) for pain. CT scans and radiographs were conducted prior to and 12 weeks after index surgery.

**Results:**

Seventy-nine patients were available for clinical evaluation at 3 ± 0.4 years follow-up. Four implants had to be revised for early loosening or infection (4.4%). Four patients had died and three patients were not able to follow the invitation for clinical assessment. Functional results in the KSS were significantly lower after navigated TKA. Operation time and incisions with navigation were significantly longer. Significantly less radiological outliers with navigation were found for coronal alignment of the femur, only.

**Conclusion:**

In this series, no beneficial effect for navigation in TKA could be shown assessing clinical data, as functional results in the presented series seemed to be lower after first generation navigated TKA. The clinical mid- to long-term value of navigation remains to be evaluated in larger patient series or meta-analyses at longer follow-up.

**Trial registration number:**

DRKS 00000430

## Background

Total knee arthroplasty (TKA) has become one of the most common and successful orthopedic interventions. Ten-year survival rates are reported to be higher than 90% in large patient series and registers [1]. Long-term failure modes include wear, loosening and infection. More than 50% of revisions are performed within two years after surgery and a common reason is component malposition [2]. Especially malrotation of components is reported to be a common source for postoperative pain and revisions. Diagnosis of malrotation is challenging since it usually requires computed tomography *(CT) *[3,4] and bony landmarks (usually the femoral epicondyles) can provoke imprecision with and without navigation [5,6].

Computer assisted orthopedic surgery (CAOS, navigation) has developed in large extent with improvement of software and computers since its first description. Navigation of TKA may be performed based on CT-scans or image-free at equal radiological results [7]. Today it finds its application in numerous orthopedic interventions. Navigation was successfully introduced into TKA to improve radiological accuracy of component position by avoiding position outliers in coronal and sagittal planes [8]. In this sense it has been proven an effective tool [9,10]. Most studies were not able to give statements on rotational alignment, since no CT scans were conducted. Choong et al. reported on mean values of femoral rotation as measured on full-leg CT scans, which were not different between conventional and navigated TKA but did not mention, if rotational outliers could be reduced [11].

Higher accuracy of component position with navigation is supposed to increase long-term survival and function of the implants. Good coronal femoral alignment was reported to allow better function after TKA. The same study reported trends for better function with good sagittal and rotational femoral alignment and good sagittal and coronal tibial alignment [12]. In some mid-term follow-up studies no functional differences were shown between navigated and conventional TKA [13-15]. On the other hand Choong et al. reported that Computer-assisted total knee arthroplasty achieved greater accuracy in implant alignment, which correlated with better knee function and improved quality of life [11].

This study was designed to assess the outcome of navigated TKA in comparison with conventional implantation. One focus was on clinical mid-term results, since numbers of publications in this field are still low. Secondly, the number of rotational outliers of both tibia and femur should be compared by pre- and postoperative CT-scans.

## Methods

In a prospectively randomized single-blinded approach, 90 patients with primary gonarthrosis were assigned to three different groups between August 2001 and December 2002. 30 patients received a conventional NexGen LPS without navigation (Group 1). Another 30 patients received a NexGen LPS with navigation (group 2) (Zimmer, Warsaw, Indiana, USA). In group 3, 30 patients underwent navigated TKA with the Stryker Scorpio PS (Stryker Howmedica Osteonics, New Jersey, USA). The study was approved by the institutional research board and the local ethics committee. It follows the Declaration of Helsinki principles.

Patients older than 50 years with primary osteoarthritis willing and able to give informed consent were included. Patients with rheumatoid arthritis, history of local infections and gonarthrosis secondary to trauma were excluded. Randomization was done with a computer generated list under supervision of the institutional research board.

Clinical outcome was assessed prior to the operation, at 12 weeks, at one year and at minimum two years after index surgery by an independent observer who had not been involved in the procedures without knowledge of the patients' assignment. The following clinical parameters were assessed: Physical examination with the Knee Society Score (KSS) [16], pain with visual analogue scale (VAS) according to Huskisson (VAS: 0 = no pain, 10 = worst imaginable pain), operation time, incision length, and length of hospital stay. Early and late complications were documented, too: bleeding, wound healing problems, deep infections, aseptic loosening, patella problems, stiffness, thrombosis and pulmonary embolism and abortion of navigation.

Radiographs and CT-scans were conducted prior to and 12 weeks after index surgery. Radiographs included the knee in two planes with a weight bearing anteroposterior view, and an axial view of the patella. The slope of the tibial plateau was assessed in the long lateral tibial view. The Scorpio prosthesis comes with a slope of 7° that is integrated into the PE inlay. Therefore, the ideal slope of the tibial resection is 0° for this implant. The NexGen implant comes with no intrinsic slope. Thus, the recommended slope of the tibial resection is 7°.

CT scans were performed over the knee joint as well as over the hip and the ankle of the same leg. This allowed assessment of the axis of the femoral neck, the transepicondylar axis, the tangent to the posterior femoral condyles, the axis of the tibial plateau and the axis of the ankle joint.

The leg axis was reconstructed from the CT data set. It allowed evaluation of the Mikulicz line and the hip-knee-ankle (HKA) angle expressing the mechanical axis of the leg. The mechanical lateral distal femur angle (mLDFA) is defined as the angle between the mechanical axis of the femur and the tangent to the distal joint line, measured on the lateral side of the knee joint [17]. The medial proximal tibia angle (MPTA) was defined as the angle between the mechanical axis of the tibia and the tangent to the tibial plateau, measured on the medial side of the knee [17]. Postop the mechanical axis of the tibia was drawn between the middle of the tibial implant plateau and the center of the ankle.

At latest follow-up another series of radiographs was performed. Radiographs and CT-scans were assessed by two independent examiners. Consensus was sought, whenever results were divergent. Following the manufacturer's instructions, 90° for postop MPTA and mLDFA were considered optimal. In analogy to studies published previously [8], radiological results were classified. For HKA, mLDFA and MPTA all results with a maximum deviation of 1° were categorized as ideal. Deviations > 3° were defined as outliers. For rotational alignment of femoral and tibial components, deviations of maximally 3° were regarded as ideal. Rotational deviations > 6° were considered as outliers as proposed by Jenny et al.[8]. CT scans and X-rays were evaluated by an independent observer, who was blinded to the clinical information. This was an orthopaedic resident, who had not been involved into the surgical procedures.

Surgery was performed following the manufacturers instructions using conventional instrument trays through a medial parapatellar approach under general anaesthesia. All patients were operated by two authors of this publication (M. P. and J. S.) in a femur-first technique using conventional cemented posterior stabilized components in all cases, leaving the patella unresurfaced.

The applied navigation system was the imageless (CT free) KneeTrac (Stryker Howmedica Osteonics, New Jersey, USA) [18]. Software version 1.0 was applied throughout the study. This represents the first navigation system generation and the first software version by this manufacturer. The Stryker KneeTrac uses cordless, battery powered infrared light emitters that can be positioned on a variety of implements. Optical tracker pins are attached to the distal femur, the proximal tibia and to the pelvic rim by rotation screws. The latter requires an additional small incision. Bony landmarks, such as the femoral epicondyles are identified using a pointer. Implant positions are displayed on a computer and allow adjustment of cutting jigs in real time when necessary.

Continuous variables were displayed as mean and SD. Categorical data were given in absolute numbers. After checking for equal distribution by applying the Kolmogorov-Smirnov test, values were analyzed by independent student's t-test for comparisons between groups. For pre- and postoperative comparisons within the groups the dependent student's t-test was applied. Analysis of outliers and optimally implanted components was done with the chi-squared test. A p value of < 0.05 was considered statistically significant. Statistical analysis was supported by using SPSS for Windows (SPSS Inc., Chicago, USA) and Microsoft Excel (Microsoft Corporation, Redmond, USA).

## Results

Ninety knee joints of 89 patients were randomly assigned to the three groups (61 women, 28 men). No significant differences between groups were found at baseline with respect to age, gender, BMI, KSS and VAS (table 1). All patients received the treatment that they were assigned to. Following the intention-to-treat principle all patients were evaluated in the group they were assigned to, even when navigation had to be abandoned.

**Table 1:** Demographics at baseline (mean ± SD) did not show significant differences in between groups

Parameter/Group	(1) NexGen Control (n = 30)	(2) NexGen navigated (n = 30)	(3) Scorpio navigated (n = 30)
**Age [years]**	69.6 ± 7.1	70.2 ± 5.9	69.2 ± 7.0

**Gender (f/m)**	18/12	22/8	22/8

**Body mass index (BMI)**	31.6 ± 5.4	30.4 ± 4.4	31.4 ± 4.9

**Knee Society Score (Knee)**	34.2 ± 14.5	34.1 ± 11.8	33.2 ± 10.6

**Knee Society Score (Function)**	49.7 ± 18.2	48.0 ± 19.8	51.2 ± 21.6

**Pain [Visual Analogue Scale 0-10]**	7.9 ± 1.8	7.9 ± 1.5	7.6 ± 1.7

Operation time and incision length were significantly longer in the two navigated groups (p < 0.001, table 2). There were no intraoperative complications. There were more cases of wound healing problems in the navigated groups (NexGen 4, Scorpio 5) than in the conventional control group (NexGen 1). The hospital stay was slightly longer in the navigated groups failing statistical significance (table 2). In three cases navigation had to be abandoned (two NexGen, one Scorpio) and TKA was finally performed in the conventional manner. This was due to system breakdowns caused by mechanical defects of the femur tracker in two cases and of unknown origin in one case. There were three cases of postoperative stiffness of the operated knee (two conventional NexGen, one navigated Scorpio), that were treated with mobilization under general anaesthesia. Chi-squared test revealed no statistical differences between groups in terms of perioperative complications (data not shown).

**Table 2:** Perioperative parameters (Mean ± SD)

Parameter/Group	(1) NexGen Control (n = 30)	(2) NexGen navigated (n = 30)	(2) Scorpio navigated (n = 30)
**Operation time [min]**	66 ± 14 *	98 ± 15	100 ± 18

**Length of Incision [cm]**	19.3 ± 2.4*	21.4 ± 2.1	21.4 ± 1.6

**Hospital stay [days]**	12.0 ± 2.1	14.2 ± 4.7	14.2 ± 6.8

At 12 weeks follow-up, 87 knees were included into clinical examination (table 3). There were no significant differences between the conventional and the navigated NexGen groups in terms of KSS and VAS. Results for KSS were significantly lower and for VAS were significantly higher in the Scorpio group (p < 0.04).

**Table 3:** Clinical results at 12 weeks follow-up (mean ± SD)

Parameter/Group	(1) NexGen Control (n = 28)	(2) NexGen navigated (n = 29)	(3) Scorpio navigated (n = 30)
**Knee society score (Knee)**	69.5 ± 16.9	74.1 ± 20.4	58.5 ± 19.5*

**Knee society score (Function)**	68.2 ± 16.3	69.3 ± 15.8	59.5 ± 20.2*

**Pain [Visual analogue scale 0-10]**	2.0 ± 1.5	2.2 ± 1.7	3.3 ± 2.3*

After one year 86 knees could be clinically evaluated. Scores in the KSS had increased in comparison with the 12-week follow-up. One conventional NexGen was explanted for deep infection, and one TKA was exchanged in a one step revision due to early loosening. There were no statistical differences between groups for the KSS knee score (group 1: 95.3 ± 3.6, group 2: 93.8 ± 4.0, group 3: 92.0 ± 5.7) nor the function score (group 1: 98.9 ± 3.9, group 2: 98.6 ± 3.8, group 3: 98.4 ± 5.8).

At latest follow-up (3.0 ± 0.4 years) four patients had died (1/1/2) of causes unrelated to the operation or the implant. Four TKA had to be explanted (1/1/2, 4.4%) due to infection or loosening. One patient was lost to follow-up and two patients were too ill to follow the invitation for clinical and radiological examination. Thus, 79 patients (87.8%) underwent clinical evaluation at latest follow-up. Again, KSS results were assessed and subdivided into the knee score (group 1: 93.7 ± 6.5, group 2: 94.0 ± 7.7, group 3: 89.5 ± 10.6) and the function score (group 1: 94.1 ± 14.3, group 2: 85.6 ± 20.5, group 3: 76.3 ± 28.8). Functional scores were significantly lower in the navigated groups (p < 0.01) while there were no differences in the knee score (p = 0.21). Between the two navigated groups there were no significant differences (p = 0.06 and 0.12) in terms of score results.

Postoperative radiological results as evaluated from CT scans and radiographs are displayed in table 4 as means and standard deviations. For none of the parameters tested there were significant differences in means.

**Table 4:** Postoperative radiological results (means ± SD): Slope in the NexGen group is aimed to be 7° and 0° in the Scorpio group (mLDFA: mechanical lateral distal femur angle, MPTA: medial proximal tibia angle)

Parameter/Group	(1) NexGen Control	(2) NexGen navigated	(3) Scorpio navigated
**Mechanical femoro-tibial axis**	0.8° ± 2.7°	1.0° ± 3.1°	-0.6° ± 2.6°

**mLDFA**	89.5° ± 2.5°	89.5° ± 1.5°	90.0° ± 1.3°

**Rotational alignment femoral component**	-0.9° ± 6.5°	0.2° ± 5.4°	-0.8° ± 4.5°

**MPTA**	89.1° ± 1.7°	88.9° ± 2.1°	90.0° ± 1.6°

**Rotational alignment tibial component**	-1.6° ± 6.1°	-0.6° ± 5.0°	-2.1° ± 4.2°

**Tibial Slope**	-4.0° ± 2.6°	-4.6° ± 2.3°	-2.2° ± 2.1°

The HKA showed less outliers for the conventional group (19.2% vs. 26%) as well as more optimally aligned legs (46.2% vs. 38%) without reaching statistical significance (p = 0.38 and p = 0.38).

Analyzing the mLDFA of the femoral components, there were significantly more outliers in the control group (p = 0.047). 10% of the components showed a deviation of > 3° and 47% a deviation between 1 and 3°. 43% of components were placed optimally (p = 0.44). In the navigated groups there were no outliers with a deviation > 3° from a straight mechanical femoral axis. 50% of the navigated NexGen and 63% of the navigated Scorpios were found to be placed optimally.

Applying the MPTA, 3.3% of the tibial components were considered as outliers in the control group and 50% were placed optimally. In the navigated NexGen group there were 12.3% outliers and 43.3% placed optimally. In the navigated Scorpio group there were 6.7% outliers and 70% considered optimal. Again differences failed to be statistically significant (p = 0.52 and p = 0.103).

**Figure 1:**
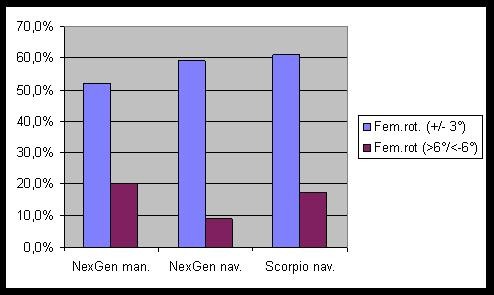
**Rotational alignment of the femoral components**.

**Figure 2:**
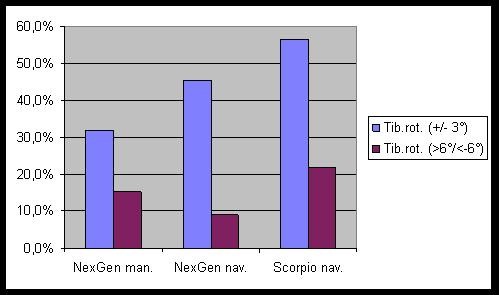
**Rotational alignment of the tibial components**.

The results of the CT-scan analysis for component rotation are displayed in figure 1 for the femur and in figure 2 for the tibia. For the femoral rotation there were slightly more optimally positioned components (59.1% and 60.9% vs. 52.0%) and some outliers less (9.1% and 17.4% vs. 20.0%) in comparison with the conventional group. These differences did not reach statistical significance (p = 0.57 and 0.805). For the tibial component differences between navigated and conventional groups were more pronounced. There were some more optimally positioned components with navigation (56.5% and 45.5 vs. 32%, p = 0.23). The numbers of rotational outliers were not significantly reduced by navigation (9.1% and 21.7% vs. 15.4%, p = 0.506).

## Discussion

For clinical mid-term results at a minimum follow-up of two years navigated knees scored significantly lower in function as assessed by the KSS. However, revision rates and survival of implants remained comparable in all groups without significant differences. Lower scores could be explained by longer incisions, more wound healing problems and longer operation time with navigation. In the early version of the Stryker navigation system three screws had to be inserted into the patients' bones: One at the pelvis, one at the distal femur and another one at the proximal tibia. To give enough room to avoid contact between the screws and the implants the skin incision had to be longer and also a second incision at the pelvis had to be made.

After 12 weeks, the navigated Scorpio group scored significantly lower in the KSS in comparison with navigated and conventional NexGen knees. This might be partially implant or procedure related. At the time of the study, the NexGen had been the primary implant at the author's institution for several years. However, also navigated NexGen scored significantly lower at mid-term in terms of function in comparison with conventional NexGen. At one year follow-up there were no significant clinical differences between conventional and navigated groups.

Incisions and operation time were longer with navigation. This did not lead to more perioperative complications such as infections or bleeding, as has been reported by other authors. Bonutti et al. reported a higher incidence of complications with navigation and attributed this to longer operative time and larger incisions [19]. In the presented cohort, there were more cases of wound healing problems in the navigated groups without differences reaching statistical significance. A longer operation time with navigation in TKA has been shown by other authors (13 - 32 minutes) [20,21]. This might mainly be due to the navigation procedure itself (placement of screws, registration of anatomic landmarks). Navigation had to be abandoned in three cases. This implicates that the surgeon has to be capable to finish the operation without help of navigation.

Analysis of component rotation from CT-scans revealed no significant differences between navigated and conventional groups. With navigation, femoral and tibial rotation was optimal more frequently, but the number of outliers could not be reduced. This is in accordance with a recent publication that showed that the virtual individual rotational position of the femoral component using a CAOS system is significantly different from its position on a postoperative CT scan [6]. In CT-less navigation the accuracy of the implantation depends on the exact identification of anatomic landmarks, which can be difficult [5]. Also there can be errors due to the fixation of the pins or the reference trackers.

For the coronal alignment, navigation managed to increase the number of optimally positioned components and to avoid outliers for the femoral side in comparison with conventional TKA as expressed in the mLDFA. This was statistically significant. For the tibial coronal positioning, results showed no significant differences. While there were more outliers and less optimal implants with navigation in the NexGen group, navigated Scorpio showed favourable results in comparison with the conventional TKA without reaching statistical significance. Less coronal and sagittal component outliers with navigation have been reported before [8,18,21-23]. A meta-analysis of alignment outcomes for navigation vs. conventional TKA including 29 studies indicated significant improvement in component orientation and mechanical axis when CAOS is used in TKA [9].

Data on rotational malalignment remains scarce. Oberst et al. reported that analysis of the rotational position of the femoral component revealed no difference between navigated and conventional TKA. Group sizes were small and there was no information on tibial rotation [24]. Chauhan et al. showed by post-operative CT significant improvement of rotational alignment of both components with navigation [21]. No differences were reported by other authors [25]. Results concerning component rotation remain inconsistent. Navigation's value for rotational alignment will have to be examined in larger patient series or by a meta-analysis.

The applied navigation system was not able to rule out all radiological outliers. The accuracy of navigation depends on several factors. Computer assisted instrumentation incorporates highly accurate measurement devices and results [26]. This might be tampered by the accuracy, of which anatomic landmarks may be defined [27-29]. Also the position of markers may accidentally change within the operation. Finally, measurements taken from x-rays and CT-scans inherit some inaccuracies, too [4]. However, it has to be considered that the KneeTrac Software version 1.0 was the first system and software version of this manufacturer. There has been a tremendous development of navigation techniques in recent years. The findings made with this system may differ from modern navigation in TKA.

The Stryker navigation system uses the epicondylar axis and the Whiteside line which were determined by the surgeon. The digitalization of the bony landmarks is one of the crucial steps in navigation. Problems of reproducibility with intraoperative termination of these landmarks have been described to especially appear with the femoral epicondyles [5,6]. The debate on how reliably these landmarks can be localized within surgery and assessed by computed tomography persists. A recent publication ruled out major inter- and intraobserver failure for the determination of femoral and tibial rotation [30].

No clinical benefits for navigation at short-term have been shown in other publications [20]. Kamat et al. showed no difference in clinical outcome measures between navigated and conventional TKA at 5 years in a large patient series. While malaligned knees showed worse clinical results they concluded that significant differences might develop at long term [13]. Other studies could not show clinical differences at 2 years [14] or at five years [15]. In one series, computer-assisted TKA achieved greater accuracy in implant alignment, which correlated with better knee function and improved quality of life [11]. Data on mid- and long-term follow-up remains scarce. In the presented series functional outcome was lower in the navigated group. More clinical studies at longer follow-up are needed to assess the value of navigation for functional outcome of TKA e. g. by means of meta-analysis.

There were no differences in patient demographics in between groups but nevertheless, there are some limitations to the presented study. Patient blinding of the randomization might have been tampered by the additional cut at the pelvic rim for one of the bone markers in the navigated group. TKA has been a successful intervention over the last decades at high patient satisfaction rates. Thus, remarkable improvement of outcome by new technologies might be hard to achieve and even harder to be proven. With 30 patients, group sizes remain small. Some of the differences discussed above might have become significant at larger patient numbers. The presented results should help to determine group sizes for future studies with bigger patient numbers for a more profound analysis e.g. of rotational alignment of components and clinical results.

To date there is little data on rotational alignment of TKA components in dependence of navigation. First generation navigation allowed slightly higher accuracy of both rotational and coronal component position, although differences failed significance for most of the assessed angles in the presented group size. However, this did not lead to superior clinical results in the applied scores. Functional results with navigation were even lower in comparison with the conventional procedure at mid-term follow-up. Key factor for the evaluation of an endoprosthetic procedure is the long-term survival of the implant. Thus, success of navigation has to be re-evaluated at a longer follow-up.

## Conclusion

It can be stated, that significantly less radiological outliers with first generation navigation were found for coronal alignment of the femur, only. At minimum two years follow-up no beneficial effect for navigation in TKA could be shown assessing clinical data, as functional results in the presented series seemed to be lower after navigated TKA. Its clinical long-term value remains to be evaluated.

## Abbreviations

BMI: Body Mass Index; CAOS: Computer Assisted Orthopaedic Surgery; CT: Computer Tomography; HKA: Hip Knee Ankle Angle; KSS: Knee Society Score; mLDFA: mechanical Lateral Distal Femoral Angle; MPTA: Medial Proximal Tibial angle; TKA: Total Knee Arthroplasty; VAS: Visual Analogue Scale.

## Competing interests

The authors declare that they have no competing interests.

## Authors' contributions

JS Analysis and interpretation of data, drafting of the manuscript. CH Acquisition of data. HK Conception and design and final approval of manuscript. MP Analysis and interpretation of data, final approval of manuscript. TE Analysis and interpretation of data, drafting of the manuscript. SF Conception and design and final approval of manuscript. TH Analysis and interpretation of data, drafting of the manuscript.

## Pre-publication history

The pre-publication history for this paper can be accessed here:

http://www.biomedcentral.com/1471-2474/12/16/prepub
